# Mobile Health for Central Sleep Apnea Screening Among Patients With Stable Heart Failure: Single-Cohort, Open, Prospective Trial

**DOI:** 10.2196/cardio.9894

**Published:** 2019-03-19

**Authors:** Roderick Willem Treskes, Arie C Maan, Harriette Florence Verwey, Robert Schot, Saskia Lambertha Maria Anna Beeres, Laurens F Tops, Enno Tjeerd Van Der Velde, Martin Jan Schalij, Annelies Margaretha Slats

**Affiliations:** 1 Department of Cardiology Leiden University Medical Center Leiden Netherlands; 2 Department of Pulmonology Leiden University Medical Center Leiden Netherlands

**Keywords:** mobile health, central sleep apnea, heart failure, prevention, screening, mobile phone

## Abstract

**Background:**

Polysomnography is the gold standard for detection of central sleep apnea in patients with stable heart failure. However, this procedure is costly, time consuming, and a burden to the patient and therefore unsuitable as a screening method. An electronic health (eHealth) app to measure overnight oximetry may be an acceptable screening alternative, as it can be automatically analyzed and is less burdensome to patients.

**Objective:**

This study aimed to assess whether overnight pulse oximetry using a smartphone-compatible oximeter can be used to detect central sleep apnea in a population with stable heart failure.

**Methods:**

A total of 26 patients with stable heart failure underwent one night of both a polygraph examination and overnight saturation using a smartphone-compatible oximeter. The primary endpoint was agreement between the oxygen desaturation index (ODI) above or below 15 on the smartphone-compatible oximeter and the diagnosis of the polygraph.

**Results:**

The median age of patients was 66.4 (interquartile range, 62-71) years and 92% were men. The median body mass index was 27.1 (interquartile range, 24.4-30.8) kg/m^2^. Two patients were excluded due to incomplete data, and two other patients were excluded because they could not use a smartphone. Seven patients had central sleep apnea, and 6 patients had obstructive sleep apnea. Of the 7 (of 22, 32%) patients with central sleep apnea that were included in the analysis, 3 (13%) had an ODI≥15. Of all patients without central sleep apnea, 8 (36%) had an ODI<15. The McNemar test yielded a *P* value of .55.

**Conclusions:**

Oxygen desaturation measured by this smartphone-compatible oximeter is a weak predictor of central sleep apnea in patients with stable heart failure.

## Introduction

Central sleep apnea is characterized by sleep-disordered breathing associated with diminished or absent respiratory effort. It is often accompanied by symptoms of tiredness, excessive daytime sleepiness, and frequent nocturnal awakening [1,2]. Central sleep apnea and Cheyne-Stokes respiratory breathing are common in patients with congestive heart failure, with a reported prevalence of 30%-50% [3]. Moreover, central sleep apnea in chronic heart failure is associated with increased mortality and reduced left ventricular function [4]. In addition, treatment of central sleep apnea with continuous positive airway pressure in cases of chronic heart failure has shown to improve left ventricular function in patients who respond to treatment [5].

Polysomnography is the gold standard for the diagnosis of central sleep apnea. However, it is a burden to the patient, as it disrupts sleep. Furthermore, the process is time consuming for technicians, as one polysomnography examination takes 2 hours to fully evaluate. The polygraph examination is an easier way of evaluating sleep-disordered breathing than full polysomnography, but the former also still disrupts normal sleep for a patient with an already reduced quality of life. Other screening methods to reduce the number of polygraphs may therefore be preferred.

Developing new screening methods, including electronic health (eHealth) apps, questionnaires, and wireless overnight pulse oximetry for patients with congestive heart failure might optimize the number of patients screened for central sleep apnea. Furthermore, it may be more patient friendly in a group of patients with an already diminished quality of life.

One possible screening method is the use of eHealth apps. Recent developments in the eHealth industry resulted in a variety of eHealth apps that claim that they can detect sleep-disordered breathing. However, most of these apps are not clinically validated. One example of an app that, according to the manufacturer, provides accurate saturation measurements is the iSpO_2_ app (Masimo Corporation, Irvine, California) [6,7]. By using the app and an oximeter, a user can record saturation, heart rate, and pulse index. Digital storage of data allows for rapid transmission and analysis, minimizing the involvement of technicians. Previous research has suggested that overnight oximetry can be used to detect obstructive sleep apnea in various patient populations [8]. However, overnight oximetry with an eHealth device has not been evaluated as a screening method for patients with stable heart failure. Therefore, this study aimed to evaluate whether overnight pulse oximetry can be used to identify patients with central sleep apnea in a population with congestive heart failure by using a validated mobile health app.

## Methods

### Patient Population

Patients with stable heart failure who visited the outpatient clinic of the Department of Cardiology at the Leiden University Medical Center were eligible for study participation if they met all inclusion and exclusion criteria. The inclusion and exclusion criteria are listed in Textbox 1. Briefly, patients who had stable heart failure according to the European Society of Cardiology guidelines [9], no history of obstructive sleep apnea or central sleep apnea, no history of ischemic or hemorrhagic stroke, and a life expectancy of more than 12 weeks (as per the physician’s discretion) were eligible.

### Study Design and Procedure

**Figure 1:**
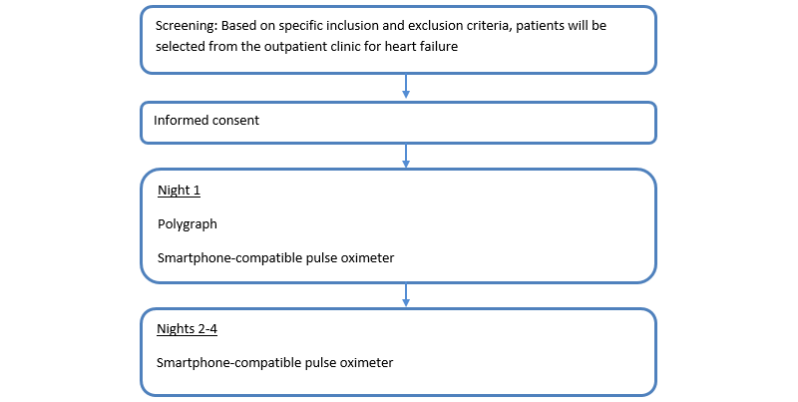
Flowchart of study procedures.

The study was a single-cohort, nonrandomized, open, prospective trial. Patients with stable heart failure were asked to participate by a treating cardiologist at a regularly scheduled heart failure outpatient clinic visit. Patients received information from a project-dedicated health care professional. If patients were willing to participate, they visited the Department of Pulmonology within 1.5 months of the outpatient clinic visit. At day 1, a project-dedicated health care professional with ample training performed the polygraph. Each patient was given a smartphone and smartphone-compatible oximeter and received oral and written instructions on their use. Patients were instructed to attach the smartphone-compatible oximeter contralateral to the hand where the polygraph was attached. During the first night, patients slept with both the polygraph and the smartphone-compatible oximeter attached. After one night, patients returned the polygraph to the hospital. On the second, third, and fourth nights, patients slept with only the smartphone-compatible oximeter attached. After the fourth night, patients returned the smartphone-compatible oximeter to the hospital. A flowchart of these events is presented in Figure 1.

### Devices

The polygraph equipment (Cidelec, Angers, France) consisted of a nasal cannula, a suprasternal sensor, thoracic and abdominal gauges, a finger pulse oximeter, a light sensor, body position sensor, and an activity sensor. The pulse oximeter has a sampling rate of 8 Hz. Both the smartphone (iPhone 5s; Apple, Cupertino, CA) and the smartphone-compatible pulse oximeter (Masimo) were provided by the hospital for the duration of the study. The smartphone-compatible pulse oximeter is worn at the fingertip and is connected with the smartphone via a wire. The pulse oximeter has a sampling rate of 1 Hz.

Devices used in this study were battery powered, electrically safe, and approved by the hospital’s Instrumentation Department.

**Inclusion and exclusion criteria.**

**Inclusion criteria:**

- Chronic heart failure according to the European Society of Cardiology guidelines [9]
- Age≥18 years

**Exclusion criteria:**

- History of obstructive sleep apnea syndrome
- History of central sleep apnea syndrome
- History of ischemic stroke
- History of hemorrhagic stroke
- History of chronic obstructive pulmonary disease
- Evidence of fluid retention at the time of study inclusion
- History of surgery under general anesthesia ≤3 months before study inclusion
- Intravenous injection of diuretics ≤1 month before study inclusion
- Unwilling to sign the informed consent form
- Life expectancy ≤12 weeks as per the physician’s discretion
- History of left ventricular assist device implantation
- Use of oxygen on a daily basis
- Pregnancy

### Data Analysis

Central sleep apnea and obstructive sleep apnea were diagnosed on the basis of the polygraph examination results in accordance with the America Association of Sleep Medicine guidelines [10]. Patients were diagnosed with sleep apnea if the polygraph showed an apnea-hypopnea index of ≥15 per hour. Sleep apnea was subsequently classified as central or obstructive sleep apnea. A patient was diagnosed with central sleep apnea if ≥50% of all apnea and hypopnea events were classified as “central.” A patient was diagnosed with obstructive sleep apnea if <50% of all apnea and hypopnea events were classified as “central.” Definitions for apnea and hypopnea events and their subdivision as central or obstructive were derived from the America Association of Sleep Medicine manual for the scoring of sleep and associated events [10]. The oxygen desaturation index (ODI) was defined as the average number of dips in saturation per hour. A cut-off value of 15 was chosen for the ODI. A dip was defined as a ≥3% decrease in saturation that lasted ≥10 seconds from the baseline saturation. The baseline saturation was determined in the hospital right after the polygraph was attached to the patient. The polygraphs were reviewed by a senior pulmonary physician with ample training who was blinded to the results of the oximeter-compatible app.

The oximeter-compatible app (Masimo) generated a comma-separated value (CSV) file, which was imported into a dedicated Matlab script (The MathWorks, Natick, MA), from which the average pulse oximeter saturation (SpO_2_), lowest SpO_2_, total percentage of time spent with a saturation <90%, and ODI were calculated. The ODI was again defined as the average number of dips in saturation per hour. A dip was defined as a ≥3% decrease in saturation that lasted ≥10 seconds from the average saturation over the 11th minute of measurement [10]. The smartphone-compatible oximeter data were analyzed by a project-dedicated professional with ample training, who was blinded to the results of the polygraph.

### Endpoints

The primary endpoint is an agreement between ODI of the smartphone-compatible oximeter and the diagnosis of the polygraph, expressed as four numbers (the number of patients who have central sleep apnea, as diagnosed by polygraph, and ≥15 dips/hour on the smartphone-compatible oximeter; the number of patients who have central sleep apnea and ≤15 dips/hour; the number of patients who do not have central sleep apnea and ≥15 dips/hour; the number of patients who do not have central sleep apnea and ≤15 dips/hour) in a 2 ⨯ 2 table.

The secondary endpoints include (1) the percentage of cases of sleep apnea (either of obstructive or central etiology) detected in the study population by the polygraph; (2) the percentage of cases of central sleep apnea detected in the study population by the polygraph; (3) agreement between the ODI, measured by the polygraph, and sleep apnea (either obstructive or central etiology) in the study population; (4) agreement between the ODI, measured by the polygraph, and central sleep apnea in the study population; (5) median difference in ODI, lowest saturation, and average saturation between the polygraph and mobile pulse oximeter; (6) sensitivity and specificity of pulse oximetry to detect central sleep apnea by saturation dips>15/hour; and (7) the percentage of patients able to use the eHealth device as instructed.

### Statistical Analysis

R (R foundation for statistical computing, Vienna, Austria) was used to perform a power calculation for the McNemar test. An alpha level of .05 and a beta level of 0.20 were chosen. Based on unpublished research by our study group, we estimated the ratio of p01/p10 (p01, false positives; p10, false negatives) to be 12, and the sum of p10 and p01 to be 0.39. This yielded a sample size of 26 patients.

SPSS version 22.0 (IBM Corp, Armonk, NY) was used for statistical analysis. Continuous variables are expressed as median with interquartile range (IQR) from the 25th to the 75th percentile.

Significance of the primary endpoint was calculated using the McNemar test. A *P* value≤.05 was considered statistically significant. A Bland-Altman plot was drafted to assess the short-term reproducibility of the ODI, with the ODI of the first night depicted on the X-axis and the difference in the ODI between the first and second night depicted on the Y-axis.

### Ethical Approval

This study was conducted in accordance to the principles of the Declaration of Helsinki (version 10, October 2013) [11] as per the Dutch Medical Research Involving Human Subjects Act [12] and Good Clinical Practice [13]. The study was approved by the hospital’s Medical Ethics Committee (P15.211). All subjects provided written informed consent before inclusion in the study. Devices used in this study were all battery powered and electrically safe. All devices were approved by the hospital’s Instrumentation Department. All devices used in this study were purchased from manufacturers. No manufacturer had any role in or influence on the design or conduct of the study, data analysis, writing of the manuscript, or the decision to submit for publication. No financial support for this study was received from any manufacturer.

## Results

### Patient Population

A total of 26 patients were included in the study. The median age was 66.4 (IQR: 62-71) years, and 92% were men. The median body mass index was 27.1 (IQR: 24.4-30.8) kg/m^2^. All patients had New York Heart Association class I (15.4%) or class II (84.6%) heart failure, and 61.5% had an ischemic cardiomyopathy. The median left ventricular ejection fraction was 34% (IQR: 24%-45%), median probrain natriuretic peptide level was 748 (IQR: 244.6-1479) ng/L, and median neck circumference was 41 (IQR: 38-44) cm. The population characteristics are summarized in Table 1.

### Polygraph

A total of 26 polygraph examinations were performed. One polygraph examination was of insufficient diagnostic quality and one polygraph examination was too short to establish a diagnosis. Both patients were not willing to undergo a second polygraph examination. Of the 24 patients that underwent a polygraph examination of diagnostic quality, 14 (58%) had sleep apnea (of either etiology); in addition, 8 of the 24 (33%) were diagnosed with central sleep apnea and 6 (25%) were diagnosed with obstructive sleep apnea (secondary endpoints 1 and 2 [see Endpoints section]). In 10 of the 24 (41%) cases, no sleep apnea was detected. The median sleep duration was 6.5 (IQR: 5.4-7.4) hours, median apnea-hypopnea index was 17 (IQR: 6.5-27.8), median ODI was 16 (IQR: 5.5-28), median number of hypopneas per night was 62 (IQR: 30.8-79.8), and median number of dips was 92.5 (IQR: 33.3-156).

**Table 1:** Baseline characteristics of the study population (N=26).

Characteristics		Value
Age (years), IQR^a^		66.4 (62.2-70.6)
Male gender, n (%)		24 (92.3)
Body mass index (kg/m^2^), median (IQR)		27.1 (24.4-30.8)
**NYHA^a^** **class, n (%)**		
	I	4 (15.4)
	II	22 (84.6)
Ischemic cardiomyopathy, n (%)		16 (61.5)
Left ventricular ejection fraction, median (IQR)		34 (23.5-45)
Pro-brain natriuretic peptide level, median (IQR)		748 (244.6-1479)
Neck circumference (cm), median (IQR)		41 (35-49)

^a^IQR: interquartile range.

^b^NYHA: New York Heart Association.

### Overnight Oximetry

**Figure 2:**
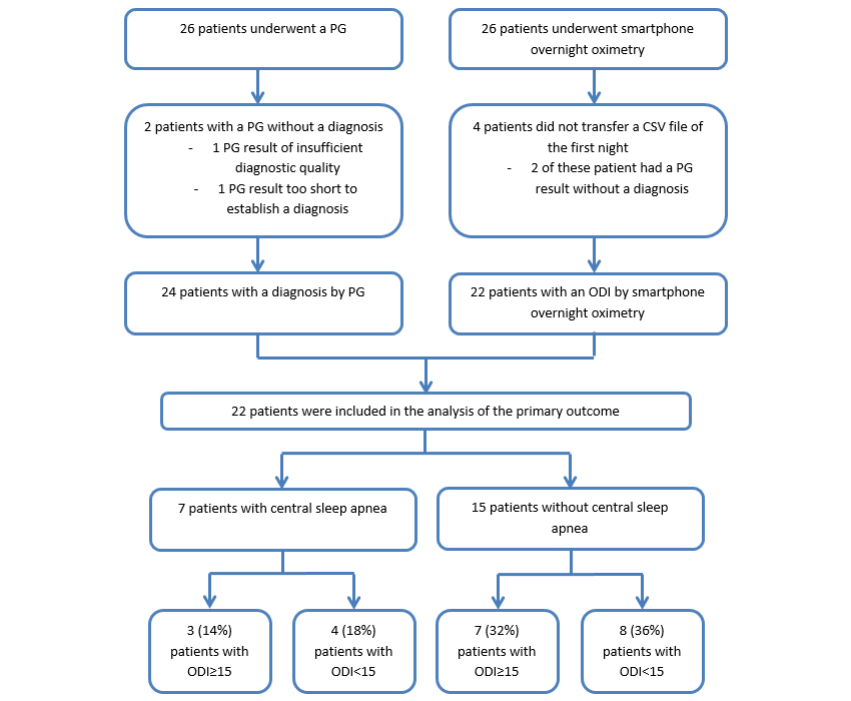
Flowchart of the results of the primary outcome. PG: polygraph; ODI: oxygen desaturation index.

All 26 participants transferred at least one CSV file containing the overnight saturation measured by the smartphone-compatible pulse oximeter. Of the 4 patients who did not transfer a CSV file of their first night (the night they also underwent the polygraph), 2 patients could not be diagnosed because the polygraph was of insufficient quality (as described above). The other 2 patients forgot to attach the smartphone-compatible pulse oximeter on their first night. Therefore, 22 patients were included in the analysis of the primary endpoint and secondary endpoints 3, 4, 5 and 6 (see Endpoints section; Figure 2). Of the 26 patients who participated, 13 (50%) were able to transfer CSV files of four consecutive nights. A total of 9 (35%) patients transferred CSV files of three nights, 1 (4%) transferred CSV files of two nights, and 3 (12%) transferred CSV files of only one night.

Of all files transferred, the median saturation was 95.7 (IQR: 94.5-96.7). The median lowest saturation was 87 (IQR: 82-90), and the median ODI was 10.1 (IQR: 2.9-20.3). The total number of dips was 75.5 (21-144.8), and total sleep time was 8.1 (6.7-9.4) hours.

### Primary Endpoint

Of the 7 (of 22, 32%) patients with central sleep apnea who were included in the analysis, 3 (14%) had an ODI≥15. The other 4 (18%) patients had an ODI<15. Of all 15 patients without central sleep apnea who were included in the analysis, 8 (36%) had an ODI<15 (Table 2). The McNemar test yielded a *P* value of .55.

### Secondary Endpoints

Of all 13 patients with sleep apnea, 6 had an ODI≥15, measured by Masimo. Of all 9 patients without sleep apnea, 5 had an ODI<15 (Table 3).

Of the 7 patients with central sleep apnea, 6 had an ODI ≥15 (measured by the polygraph). Of the patients without central sleep apnea, 9 had an ODI<15 (Table 4). Of all 13 patients with sleep apnea (of either etiology), 12 had an ODI≥15 (measured by the polygraph). All 9 patients without sleep apnea had an ODI<15 (Table 5).

The sensitivity of the ODI for the detection of central sleep apnea is 43%, and the specificity is 53%. The positive predictive value is 30%, and the negative predictive value is 67%. The sensitivity of the polygraph is 86%, and the specificity is 53%.

### Difference Between the Polygraph and Mobile Pulse Oximeter

The median difference in ODI was 2.1 indices higher than that measured with the polygraph (IQR: –4.3 to 14.3). The smartphone-compatible oximeter yielded a median saturation of 3.1 (IQR: 2.6-4.1) percentage points higher than the that measured by the polygraph. There was no difference in the median lowest saturation measured by both devices (both 83%).

**Table 2:** Number of patients with central sleep apnea (yes/no) and an ODI of ≥15 or <15 (as measured by the smartphone-compatible oximeter).

Oxygen desaturation index measured by mobile pulse oximeter	Central sleep apnea present, n (%)	Central sleep apnea absent, n (%)	Total, n (%)
≥15	3 (14)	7 (32)	10 (46)
<15	4 (18)	8 (36)	12 (54)
Total	7 (32)	15 (68)	22 (100)

**Table 3:** Number of patients with sleep apnea (yes/no) and an ODI of ≥15 or <15 (as measured by the smartphone-compatible pulse oximeter).

Oxygen desaturation index measured by smartphone-compatible oximeter	Sleep apnea present, n (%)	Sleep apnea absent, n (%)	Total, n (%)
≥15	6 (27)	4 (18)	10 (45)
<15	7 (32)	5 (23)	12 (55)
Total	13 (59)	9 (41)	22 (100)

**Table 4:** Number of patients with central sleep apnea (yes/no) and an ODI of ≥15 or <15 (as measured by the polygraph).

Oxygen desaturation index measured by polygraph	Central sleep apnea present, n (%)	Central sleep apnea absent, n (%)	Total, n (%)
≥15	6 (27)	6 (27)	12 (54)
<15	1 (5)	9 (41)	10 (46)
Total	7 (32)	15 (68)	22 (100)

**Table 5:** Number of patients with sleep apnea (yes/no) and an ODI of ≥15 or <15 (as measured by the polygraph).

Oxygen desaturation index measured by polygraph	Sleep apnea present, n (%)	Sleep apnea absent, n (%)	Total, n (%)
≥15	12 (54)	0 (0)	12 (54)
<15	1 (5)	9 (41)	10 (46)
Total	13 (59)	9 (41)	22 (100)

## Discussion

### Principal Findings

This study investigated the use of a smartphone-compatible oximeter to measure the ODI for the detection of central sleep apnea in patients with stable heart failure. Oxygen desaturation, when measured by an eHealth device, appeared to be a weak predictor of central sleep apnea in patients with stable heart failure. In addition, in this elderly group of patients, the correct use of this eHealth device was only achieved by 50% of patients. On the other hand, ODI measured by the polygraph might be a good predictor of sleep apnea of any etiology in patients with stable heart failure. We found that 58% of participating patients had sleep apnea. Of all patients, 33% had central sleep apnea and 25% had obstructive sleep apnea. These percentages are lower than the prevalence reported by Oldenburg et al [14]: In a screening study of 700 patients, they showed that 70% of patients had sleep apnea, of which 40% had central sleep apnea and 30% had obstructive sleep apnea. This difference may be explained (at least partly) by the relatively small sample size, but possibly also by the differences in severity of heart failure in these patients.

In our study, a median number of 92.5 dips was observed. This number was significantly higher than that in a previous study [15] of 12 patients with heart failure, which reported 4 dips per patient per night. This difference may largely be due to the difference in definition of a “dip.” Davies et al [15] defined a dip as “a fall of >4% in oxygen saturation from a stable baseline that lasted >30 seconds,” while in the current study, a dip was defined as a ≥3% decrease lasting 10 seconds. This criterion was necessary to define dips equally between the polygraph software and our smartphone-compatible oximeter.

### Oxygen Desaturation Index as a Potential Screening Tool for Central Sleep Apnea

This study showed that the ODI, measured by either the polygraph or smartphone-compatible oximeter, correlates poorly with the diagnosis of central sleep apnea. The McNemar test yielded a nonsignificant *P* value of .55. However, there was a good correlation between the ODI measured by the polygraph and the diagnosis of sleep apnea (of either etiology). We acknowledge that the study was not powered on this outcome. Furthermore, hypopneas in polygraphs are scored based on desaturation events. Therefore, the diagnosis of sleep apnea is partially dependent on the ODI. However, the strong outcome of 0 false positives and 1 false negative indicates that the ODI might indeed be a good screening method and should be investigated in further research.

### Implications for Clinical Practice

Our study found that 58% of patients with stable heart failure had either form of sleep apnea (central or obstructive etiology). Both obstructive and central sleep apnea are associated with higher mortality and lower quality of life in patients with stable heart failure [4]. Therefore, early diagnosis is of paramount importance. However, screening for obstructive sleep apnea or central sleep apnea is not recommended by current guidelines, but with such a high prevalence, routine screening of patients should be considered. Perhaps, screening should not focus on the distinction between obstructive sleep apnea and central sleep apnea, as both have clinical implications [9]. Since ODI correlates well with sleep apnea of any etiology, research on easy-to-use overnight oximetry eHealth devices for sleep apnea screening among patients with stable heart failure is necessary.

### Electronic Health Use in a Population With Heart Failure

In this study, patients were asked to attach, record, and email the overnight saturations themselves. Instructions about the use of the smartphone, the Masimo patch, and the emailing of the CSV files were given after the polygraph was attached to the patient. However, of all patients, 13 were unable to transfer four CSV files. These results should be seen as hypothesis generating, but also indicate that when conducting a study in an older and vulnerable population, the eHealth system should be tailored to the patient population. Furthermore, time spent in patient education of the eHealth system should not be underestimated.

### Differences in Saturation Measured by the Polygraph and the Mobile Pulse Oximeter

Our results showed some significant differences in the predictive value of the ODI for both sleep apnea of any etiology and central sleep apnea, median ODI, and median average saturation between the polygraph and the smartphone-compatible pulse oximeter. There are several explanations for this phenomenon. First, it is unclear how motion artefacts influenced our results. Motion during sleep and movements of the fingers might result in different results from the oximeter of the polygraph, which has been designed specifically for overnight saturation measurement. Second, patients attached the smartphone-compatible oximeter themselves at home. Although instructions were given in the hospital, it is uncertain whether patients attached the device correctly. Improper placement usually gives no signal and therefore no saturation in the CSV file. On the other hand, slight improper placement might result in improper values in the CSV file.

### Limitations

This study has some limitations that have affected its results. Unfortunately, in two patients, it was not possible to obtain a diagnosis from the polygraph. These two patients were not willing to undergo a second polygraph. However, given the numbers of the primary endpoint and a relatively high *P* value of .55, it is unlikely that two extra patients would have changed the data significantly. Furthermore, some patients could not deal with the smartphone technology given, despite ample instructions. As a consequence, 13 patients were unable to record their overnight saturation for four consecutive nights. Lastly, we did not perform polygraph examinations in healthy volunteers. Therefore, a comparison of overnight oximetry with aged-matched healthy volunteers is lacking, although this has been previously reported in the literature [16].

### Conclusions

Oxygen desaturation, when measured by the eHealth oximeter tested in this study is a weak predictor of central sleep apnea in patients with stable heart failure. The ODI, when measured by a validated device, might be a good predictor of sleep apnea of any etiology in patients with stable heart failure. This study also corroborated the high prevalence of sleep apnea in patients with stable heart failure. Therefore, more research on screening for sleep apnea in patients with stable heart failure is warranted, which might be possible by using validated overnight oximetry, but must be easy to perform in this type of elderly patient group.

## Abbreviations

CSV: comma-separated value
eHealth: electronic health
IQR: interquartile range
NYHA: New York Heart Association
ODI: oxygen desaturation index
SpO_2_: pulse oximeter saturation
